# Post-licensure safety surveillance of 9-valent human papillomavirus vaccine using the vaccine adverse event reporting system, 2014–2024

**DOI:** 10.3389/fpubh.2026.1724482

**Published:** 2026-01-20

**Authors:** Jie-Hai Chen, Ming Chen, Zhi-Yong Wu, Qing-Ming Luo, Yuan-Yan Tu

**Affiliations:** 1 Department of Anesthesiology, Dongguan Maternal and Child Health Care Hospital, Dongguan, Guangdong, China; 2 Department of Gynecology, Dongguan Maternal and Child Health Care Hospital, Dongguan, Guangdong, China; 3 Dongguan Maternal and Child Health Care Hospital, Dongguan, Guangdong, China

**Keywords:** 9-valent human papillomavirus vaccine (9vHPV), disproportionality analysis, vaccination, vaccine adverse event reporting system (VAERS), vaccine safety

## Abstract

**Background:**

On December 10, 2014, the Food and Drug Administration (FDA) licensed the 9-valent human papillomavirus vaccine (9vHPV) for prevention of HPV-related cancers and genital warts. This study aimed to summarize and characterize the first decade of post-licensure surveillance reports of 9vHPV submitted to the Vaccine Adverse Event Reporting System (VAERS).

**Methods:**

We analyzed VAERS reports following 9vHPV administration in the U.S. during December 2014 through December 2024. Disproportionality analysis was conducted using the reporting odds ratio (ROR) to identify potential safety signals. Reports were categorized by sex, age, seriousness, and clinical outcomes.

**Results:**

The VAERS received 23,499 reports following administration of 9vHPV: 46.7% were from females, 25.7% from males, and 27.6% with unreported sex. Overall, 92.5% of reports were nonserious. Syncope, dizziness, loss of consciousness and pallor were most common AEs among nonserious reports. Headache, dizziness, pain and syncope were commonly reported serious AEs. Disproportionality analysis identified six MedDRA PTs that were disproportionately reported following 9vHPV vaccination: anaphylactic shock, postural orthostatic tachycardia syndrome (POTS), dizziness postural, complex regional pain syndrome (CRPS), premature menopause and acute disseminated encephalomyelitis (ADEM). Deaths (*N* = 57) were rare and most lacked sufficient medical documentation to establish causality.

**Conclusion:**

The safety profile of 9vHPV over its first decade of use remains consistent with pre-licensure data, with most AEs being nonserious and self-limiting. Disproportionality analysis identified potential safety signals warranting further investigation but did not confirm causality. Continuous surveillance is necessary to further evaluate these rare events and ensure the ongoing safety of 9vHPV.

## 1 Introduction

The 9-valent human papillomavirus vaccine (9vHPV) (Gardasil 9, Merck & Co., Inc.) was approved by the U.S. Food and Drug Administration (FDA) in December 2014 for use in females aged 9 through 26 years and males aged 9 through 15 years for the prevention of cancers and genital warts caused by the targeted HPV types (1). In February 2015, the Advisory Committee on Immunization Practices (ACIP) recommended routine vaccination with 9vHPV for both males and females at age 11 or 12 years, with catch-up vaccination recommended for all persons through age 26 years (2). This recommendation aims to provide protection before potential exposure to HPV, with the vaccine demonstrating high efficacy in preventing HPV-related diseases within these age groups. In 2018, the FDA expanded the indication to include women and men aged 27 through 45 years, reflecting its broad public health impact (3). Thus, 9vHPV is licensed for use and recommended to protect individuals from HPV-associated cancers and warts from childhood through mid-adulthood.

9vHPV targets nine HPV types (6, 11, 16, 18, 31, 33, 45, 52, and 58), which are responsible for approximately 90% of cervical cancers and a significant proportion of other anogenital and oropharyngeal cancers (4). Pre-licensure clinical trials demonstrated its efficacy and safety, with injection site reactions and headache being the most commonly reported adverse events (AEs) (3). However, pre-licensure studies are limited in their ability to detect rare AEs due to sample size constraints and controlled study conditions.

Although pre-licensure studies demonstrated the favorable safety profile of 9vHPV, the most common adverse reactions comprised local injection site reactions such as pain, swelling and erythema, alongside systemic symptoms including headache and fever (2). Nevertheless, serious and rare vaccine-associated adverse events such as postural orthostatic tachycardia syndrome (POTS) and primary ovarian insufficiency (POI) continue to raise concerns among healthcare providers, researchers and the general public (5, 6).

Post-licensure safety surveillance is therefore critical to identify and characterize rare or delayed AEs. The Vaccine Adverse Event Reporting System (VAERS) (7), a national passive surveillance system co-administered by the Centers for Disease Control and Prevention (CDC) and the FDA, serves as a key tool for monitoring vaccine safety. VAERS collects spontaneous reports of AEs following vaccination, enabling the detection of potential safety signals and the generation of hypotheses for further investigation. However, due to underreporting and non-standardized reporting practices, VAERS signals indicate only temporal association, not causation, and necessitate confirmatory studies for interpretation. This study aims to summarize and characterize the first decade of post-licensure safety reports of 9vHPV submitted to VAERS to supplement knowledge about its safety profile.

## 2 Methods

### 2.1 Data source

VAERS is a national passive and/or spontaneous surveillance system that keeps track on the post-licensure safety of vaccines in the United States (7). It accepts reports from healthcare providers, vaccine manufacturers, and the public, including vaccine recipients and their caregivers. Reports include demographic information, vaccine details, and descriptions of AEs, which are coded using the Medical Dictionary for Regulatory Activities (MedDRA). MedDRA is a medically validated, internationally standardized terminology (8). A VAERS report may be assigned one or more MedDRA preferred terms (PTs). PT is not necessarily a medically confirmed diagnosis. According to the US Code of Federal Regulations, AEs that lead to hospitalization, prolonged hospitalization, permanent disability, life-threatening illness, congenital anomaly or birth defect, and death are classified as serious (9). In our study, if AE described met any of the above criteria, the report would be categorized as a serious report. For example, the majority of dizziness and syncope reports were categorized as non-serious AEs. However, a small proportion of these events that met the criteria were classified as serious AEs. Given that reports filed to VAERS indicate that AEs happened after vaccination rather than implying causation, VAERS data should be read cautiously.

We included reports of 9vHPV vaccinees in the United States received in the VAERS database from December 1, 2014, through December 31, 2024. The database is publicly accessible and available for free download from the official website,^1^ thereby supporting data transparency and study reproducibility. Records corresponding to GARDASIL 9 were extracted based on the vaccine name field and subsequently retained for further analysis. VAERS is used for routine monitoring as a public health function, and its data is publicly available, de-identified, and anonymized; therefore, it is not subject to review by the institutional review board.

### 2.2 Clinical review

For the descriptive analysis and data mining, all reports following 9vHPV administration were included, regardless of concomitant vaccines, to provide a comprehensive overview of the reported safety profile in real-world practice. For the in-depth clinical review of specific conditions, we adopted a primary approach focusing on reports where 9vHPV was administered alone. We conducted a comprehensive review of all reports describing deaths following 9vHPV vaccination, as well as reports of serious AEs associated with 9vHPV administration alone, with medical records examined whenever available. For each report, we identified the primary event that triggered the report and categorized it according to the MedDRA System Organ Class (SOC) and PT. Additionally, we scrutinized reports and accompanying medical records for prespecified conditions, including Guillain-Barre syndrome (GBS), anaphylaxis, postural orthostatic tachycardia syndrome (POTS), complex regional pain syndrome (CRPS), primary ovarian insufficiency (POI) (also known as premature menopause), acute disseminated encephalomyelitis (ADEM), transverse myelitis and chronic inflammatory demyelinating polyneuropathy (CIDP) (Supplementary Table S1). Where possible, we applied case definitions developed by the Brighton Collaboration for the assessment of GBS and anaphylaxis (10, 11).

### 2.3 Data mining

The study employed the reporting odds ratio (ROR) as data mining algorithm to evaluate the distribution of vaccine-event pairs. The ROR is a disproportionality measure commonly used in pharmacovigilance to detect signals of potentially disproportionate reporting within spontaneous reporting systems like VAERS. The ROR was calculated as the ratio of the odds of reporting a specific adverse event following 9vHPV vaccination compared to the odds of reporting the same event following all other vaccines in VAERS.

The ROR was calculated using a 2 × 2 contingency table:

**Table:** 

	Reports with target AE	Reports without target AE
Vaccine of interest (9vHPV)	a	b
All other vaccines	c	d


ROR=(a/b)/(c/d)

The lower limit of the 95% confidence interval (CI) for the ROR is greater than 1 and the number of AE reports is ≥3, which would be considered as a positive signal (12). Higher ROR values indicate stronger disproportion seems to be and higher probability of AEs.

## 3 Results

### 3.1 Descriptive analysis

**Figure 1:**
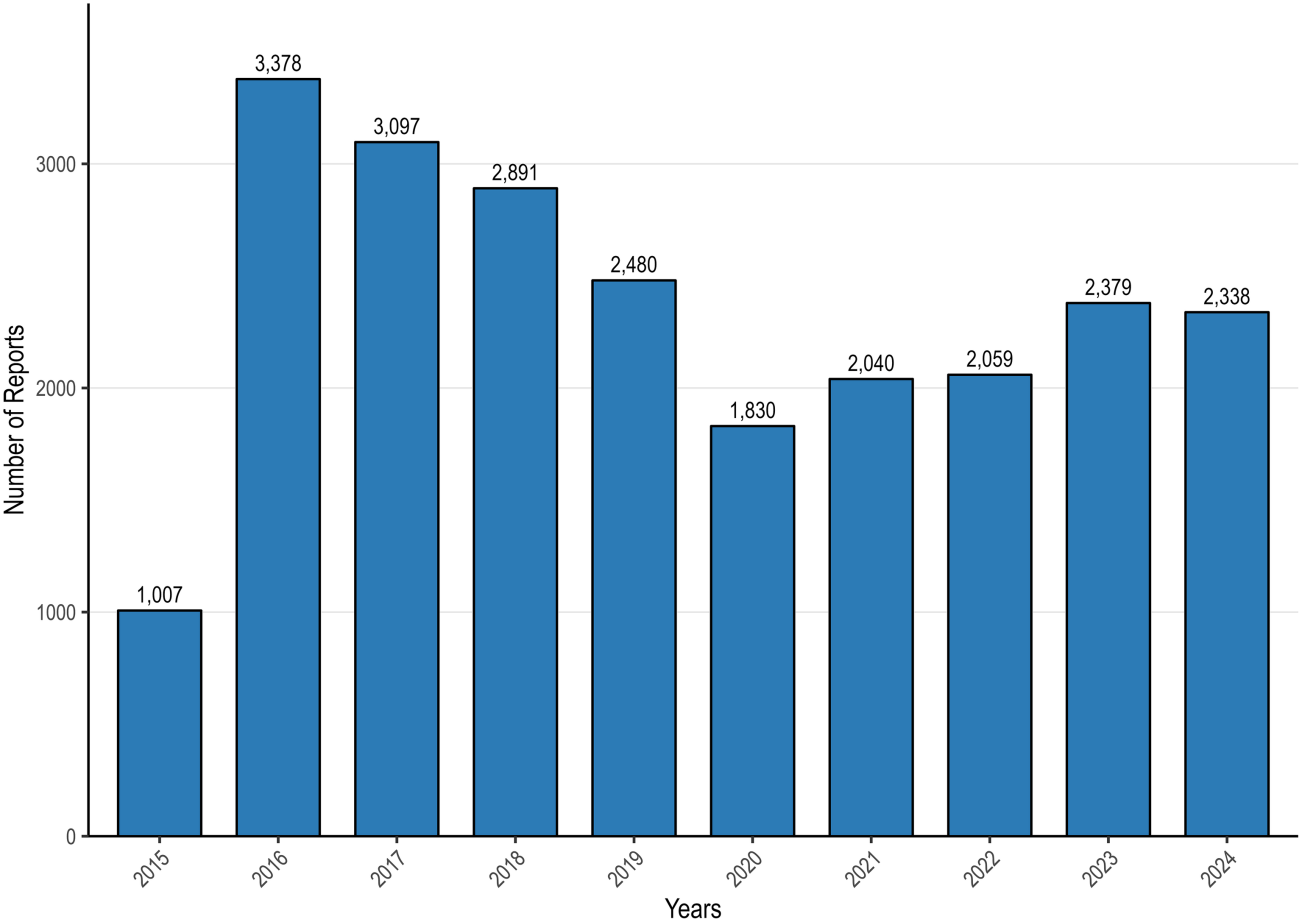
Annual reports of 9vHPV adverse events in VAERS (2014–2024). *Y*-axis represents number of reports, *X*-axis represents years.

**Figure 2:**
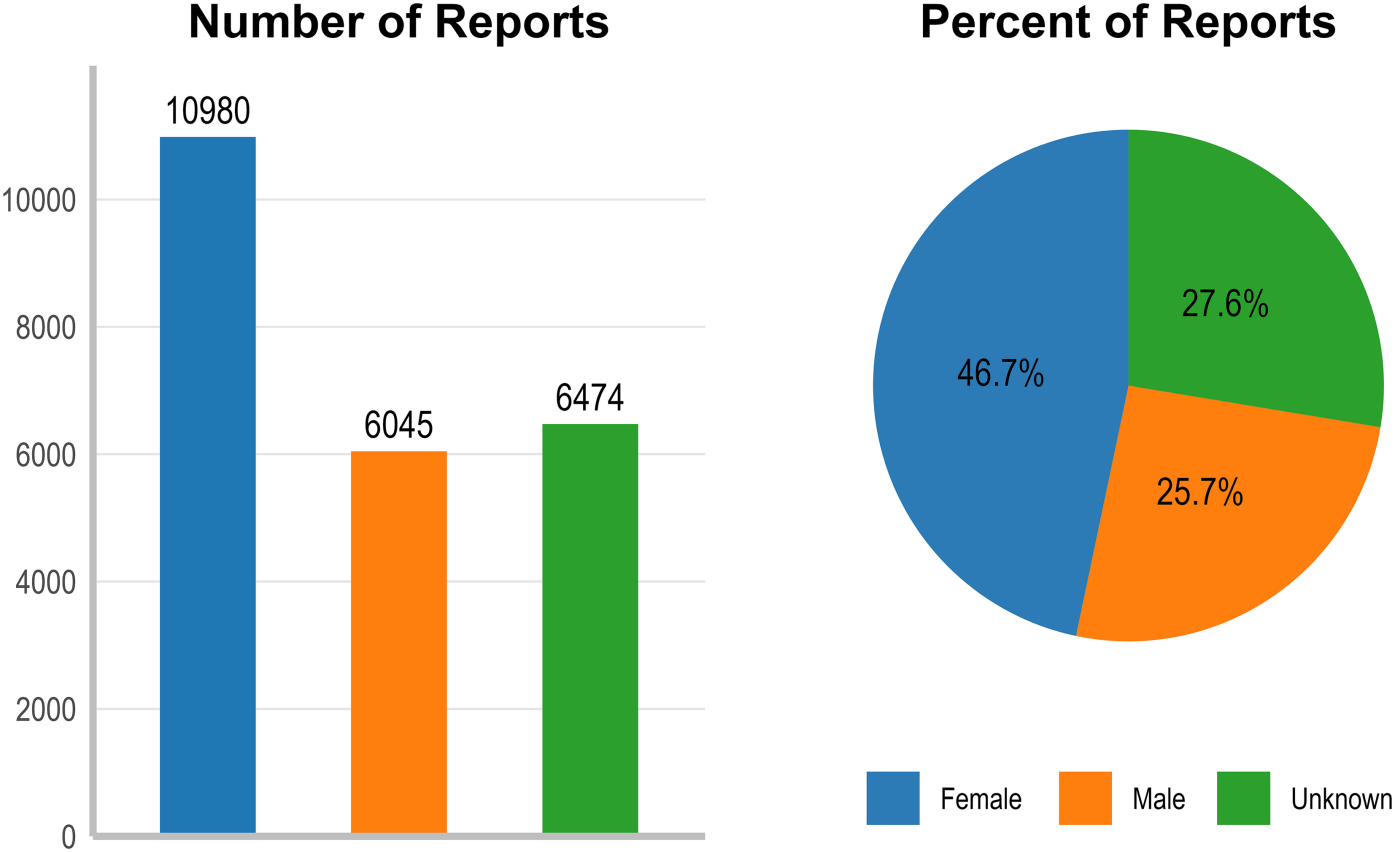
Characteristics of VAERS reports following 9vHPV (2014–2024).

**Figure 3:**
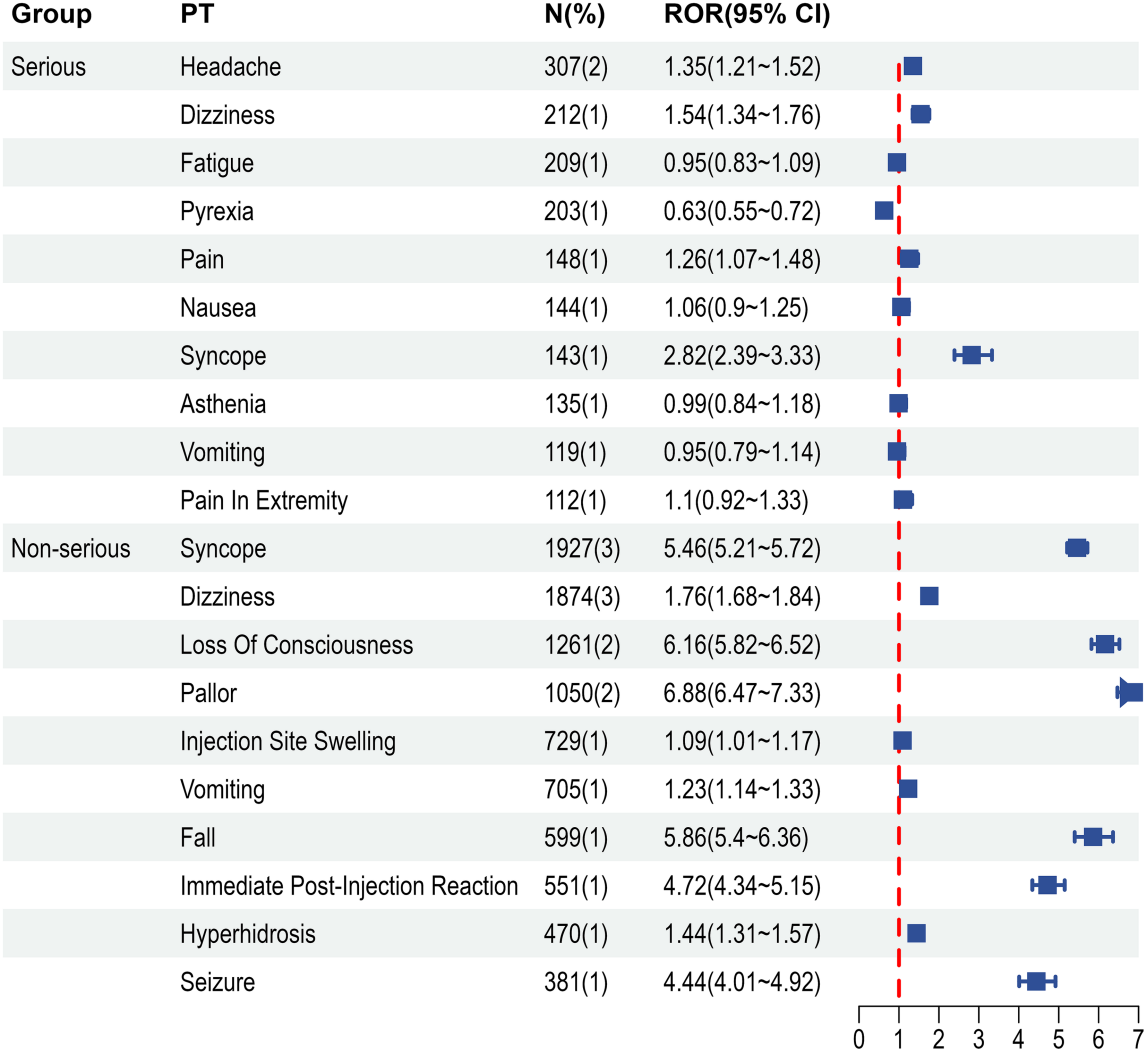
Most frequent MedDRA preferred terms for serious and non-serious reports following 9vHPV, 2014–2024. PT, preferred term; N, number of reports; ROR, reporting odds ratio.

During the analytic period, VAERS received 23,499 US reports after vaccinated with 9vHPV. Figure 1 shows the number of 9vHPV reports per year. Of these reports, 10,980 (46.7%) were from females, 6,045 (25.7%) were from males, and 6,474 (27.6%) did not report sex (Figure 2). Table 1 presents reports categorized by sex, age, adverse event onset, seriousness and fatal outcome. Overall, 92.5% of reports were nonserious. Of these, nearly 46% of the reports concerned females aged 9–17 years. Age ranges outside the recommendations were rarely reported, at 2% for those younger than 9 years and 1% for those older than 45 years. The median age of vaccine recipients was 14 years (range 0 to 97 years). The median time from 9vHPV vaccination to AEs symptom onset was 0 days. 9vHPV was administered alone in 70.6% of reports. The most commonly reported symptoms in non-serious reports were syncope, dizziness, loss of consciousness and pallor. Headache, dizziness, fatigue, pyrexia and pain were commonly reported AEs in serious reports (Figure 3).

**Table 1:** Characteristics of VAERS reports following 9vHPV administration, 2014–2024.

Report characteristics	<9 yr.	9–17 yr.	18–45 yr.	>45 yr.	Unknown	All ages
	Total = 524	Total = 10,785	Total = 3,818	Total = 229	Total = 8,143	Total = 23,499
	*N* (%)	*N* (%)	*N* (%)	*N* (%)	*N* (%)	*N* (%)
Sex						
Female	186 (35.5)	5,613 (52.0)	2,768 (72.5)	131 (57.2)	2,282 (28.0)	10,980 (46.7)
Male	163 (31.1)	4,185 (38.8)	834 (21.8)	76 (33.2)	787 (9.7)	6,045 (25.7)
Unknown	175 (33.4)	987 (9.2)	216 (5.7)	22 (9.6)	5,074 (62.3)	6,474 (27.6)
Age (years)						
Mean age (SD)	2.68 (2.70)	13.1 (2.12)	25.6 (7.45)	56.0 (9.37)	/	16.5 (8.79)
Median age [Min, Max]	1.25 [0, 8.00]	12.0 [9.00, 17.0]	24.0 [18.0, 45.0]	53.0 [46.0, 97.0]	/	14.0 [0, 97.0]
Adverse event onset (days)						
Mean (SD)	3.4 (37)	11.2 (80)	17.5 (114)	8.9 (45)	35.8 (163)	16.9 (106)
Serious/non-serious status						
Non-serious	520 (99.2)	9,910 (91.9)	3,391 (88.8)	216 (94.3)	7,691 (94.4)	21,728 (92.5)
Serious	4 (0.8)	875 (8.1)	427 (11.2)	13 (5.7)	452 (5.6)	1771 (7.5)
Deaths	0 (0)	15 (0.1)	9 (0.2)	0 (0)	33 (0.4)	57 (0.2)
Received 9vHPV alone	402 (76.7)	5,763 (56.4)	2,844 (74.5)	193 (84.3)	7,390 (90.8)	16,592 (70.6)

### 3.2 Clinical review

#### 3.2.1 Death

**Figure 4:**
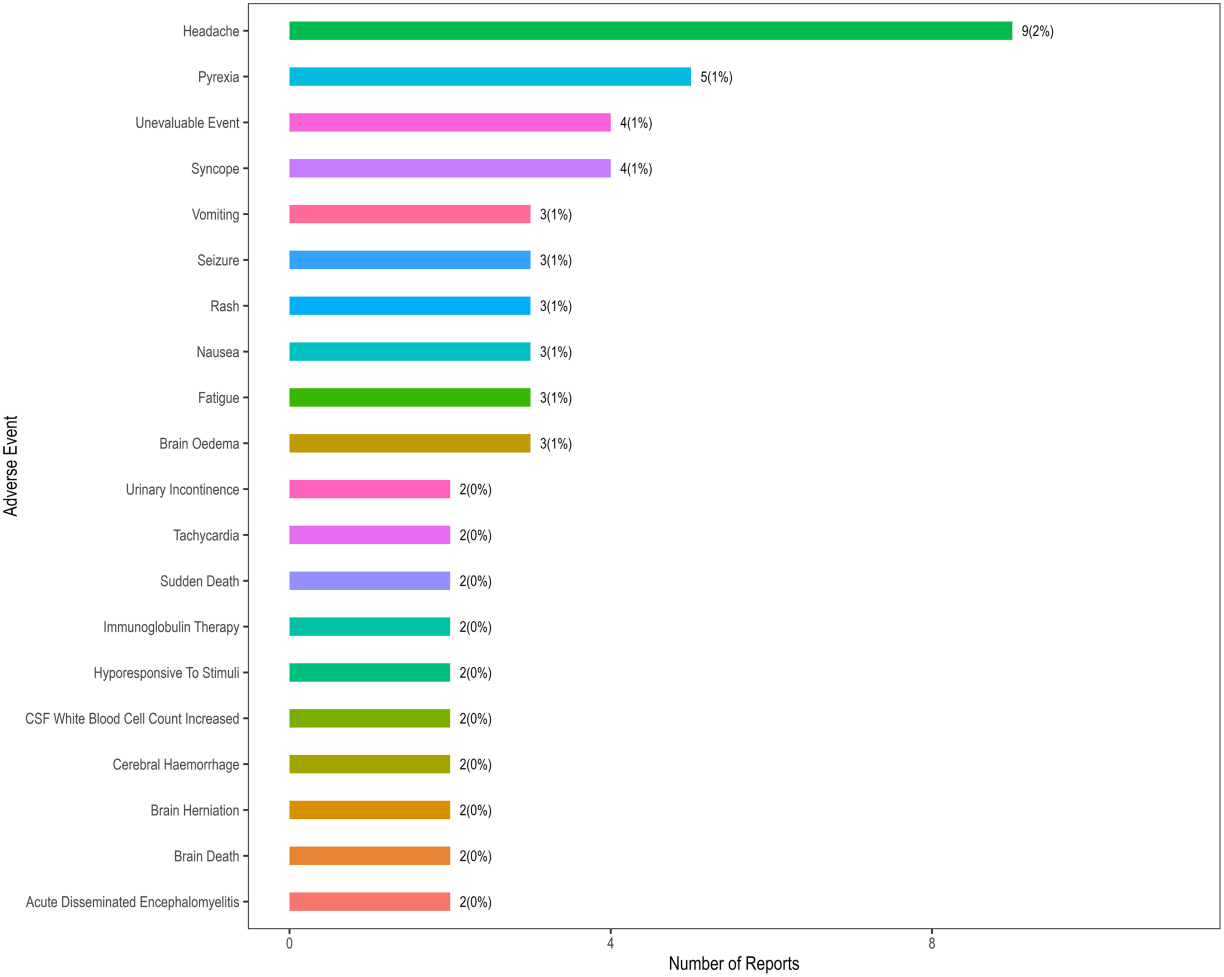
Most frequent MedDRA preferred terms for death reports after 9vHPV in VAERS, 2014–2024. *Y*-axis represents adverse event, *X*-axis represents number of reports.

Following receipt of 9vHPV, 57 deaths were reported to VAERS. Of these, 15 were aged 9–17 years, 9 were aged 18–45 years, and 33 were of unknown age. 43 had recorded cause of death or medical condition information. The quality of documentation was generally poor: in half of the reports, the cause of death was not specified and relied on speculation or vague descriptions. 30% were based on non-medical sources, such as social media, consumer reports and other sources, only 20% were verified by autopsy or medical records. A review of the narrative descriptions and MedDRA-coded terms within these reports revealed that headache (9 reports) and pyrexia (5 reports) were the most frequently documented symptoms prior to death (Figure 4). It is crucial to interpret this finding with caution: headache and pyrexia are common, non-specific symptoms that may represent prodromal manifestations of various severe underlying conditions (e.g., infections, cardiovascular events) rather than being directly causative. Even though headache and pyrexia are typically seen as transient and non-life-threatening, their observed frequency in these reports highlights the need to investigate their potential role as prodromal indicators of severe underlying pathological mechanisms in rare instances. After comprehensive review, the vast majority of reports lacked sufficient medically verified information to establish a causal link between 9vHPV vaccination and death.

#### 3.2.2 Serious reports

We analyzed serious reports after 9vHPV given alone. Table 2 lists the SOCs and the most commonly reported medical conditions described by PTs following 9vHPV vaccination alone. The most common SOC was nervous system disorders, including diagnoses of headache (221 reports), dizziness (159 reports), syncope (106 reports), hypoaesthesia (81 reports), seizures (79 reports), paraesthesia (73 reports) and loss of consciousness (68 reports). The second most common SOC was general disorders and administration site conditions, including 155 reports of fatigue, 150 reports of pyrexia, 111 reports of pain, and 105 reports of asthenia. The third highest ranking SOC was musculoskeletal and connective tissue disorders. More details of other common SOCs and PTs are given in Table 2 and Supplementary Table S2.

**Table 2:** Medical conditions for serious reports^a^ following 9vHPV given alone, 2014–2024.

Medical conditions	*N* (%)
Nervous system disorders	1,670 (14.76)
Headache	221
Dizziness	159
Syncope	106
Hypoaesthesia	81
Seizure	79
Paraesthesia	73
Loss of consciousness	68
General disorders and administration site conditions	1,399 (12.37)
Fatigue	155
Pyrexia	150
Pain	111
Asthenia	105
Musculoskeletal and connective tissue disorders	670 (5.92)
Pain in extremity	90
Arthralgia	82
Muscular weakness	75
Myalgia	49
Gastrointestinal disorders	556 (4.91)
Nausea	106
Vomiting	88
Abdominal pain	61
Skin and subcutaneous tissue disorders	531 (4.69)
Rash	69
Erythema	47
Pruritus	39
Urticaria	33
Henoch-schonlein purpura	23
Psychiatric disorders	320 (2.83)
Anxiety	38
Insomnia	26
Depression	24
Sleep disorder	16
Confusional state	15
Infections and infestations	251 (2.22)
Encephalitis	14
Nasopharyngitis	11
Pneumonia	9
Myelitis	8
Urinary tract infection	8
Respiratory, thoracic and mediastinal disorders	245 (2.17)
Dyspnoea	69
Oropharyngeal pain	14
Cough	14
Epistaxis	12
Pulmonary mass	11
Cardiac disorders	212 (1.87)
Postural orthostatic tachycardia syndrome	47
Palpitations	32
Tachycardia	28
Myocarditis	13
Sinus tachycardia	10
Injury, poisoning and procedural complications	209 (1.85)
Reproductive system and breast disorders	203 (1.79)
Amenorrhoea	19
Eye disorders	177 (1.56)
Vascular disorders	148 (1.31)
Immune system disorders	123 (1.09)
Anaphylactic shock	14
Blood and lymphatic system disorders	129 (1.14)
Metabolism and nutrition disorders	123 (1.09)
Renal and urinary disorders	78 (0.69)
Ear and labyrinth disorders	62 (0.55)
Neoplasms benign, malignant and unspecified (incl cysts and polyps)	58 (0.51)
Endocrine disorders	55 (0.49)
Hepatobiliary disorders	35 (0.31)
Congenital, familial and genetic disorders	12 (0.11)

a Serious reports are those describing death, life-threatening illness, hospitalization or prolongation of existing hospitalization, congenital anomaly or birth defect, permanent disability.

Within each System Organ Class (SOC), only the most frequently reported Preferred Terms (PTs) are listed.

#### 3.2.3 Guillain-Barre syndrome (GBS)

We identified 42 reports of possible GBS (10): 30 were female, 10 were male, and 2 did not report gender. Out of these 42 reports, 28 either lacked enough information to make a determination or did not fit the Brighton Collaboration criteria for GBS diagnosis. Of the remaining 14 reports, 7 met the case definition of GBS according to the Brighton Collaboration criteria (2 level 1, 3 level 2, 5 level 3), and 4 described GBS-like illnesses that could not be categorized, mainly due to incomplete medical records. Female cases predominated, accounting for 78.6% (11/14) of the 14 reports. Three reports documented respiratory infectious diseases, with two cases preceding the onset of GBS symptoms. 9vHPV was given alone in 10 (71.4%) reports. The median latency period between vaccination and GBS symptoms onset was 5 days (range 1–31 days).

#### 3.2.4 Anaphylaxis

We identified 89 reports of possible anaphylaxis (11): 71 were female, 15 were male, and in 3, sex was not specified. In 62 reports, 9vHPV was given alone; the remaining reports involved concomitant vaccination with other vaccines, including TDAP (*n* = 13), meningococcal conjugate (*n* = 9), 4vHPV (*n* = 5), hepatitis A (*n* = 4), influenza (*n* = 3), COVID19 (*n* = 2) and varicella (*n* = 1). A history of non-anaphylactic hypersensitivity reactions to environmental allergens, latex, food, or medications was present in four of these reports. 20 reports met Brighton Collaboration criteria Level 1, 25 met Level 2 and 12 met Level 3. Of the remaining 32 reports, 7 did not meet the Brighton Collaboration criteria and 25 had critical data gaps that precluded determination of anaphylaxis. Among 74 reports with documented onset interval, 67 (90.5%) manifested symptoms within 24 h post-vaccination administration.

#### 3.2.5 Postural orthostatic tachycardia syndrome (POTS)

We identified 136 reports of POTS (13), among which 66 cases (48.5%) meet the clinical diagnostic criteria, while the remaining cases either do not meet the criteria or lack sufficient detail to confirm a POTS diagnosis. Of the 66 confirmed reports, 60 were in females, with a median age of 14 years (range 11–41 years). The median onset interval from the day of vaccination to symptom onset was 22 days (range 0–458 days). Comorbid conditions most frequently reported included dysautonomia, mast cell activation syndrome, chronic fatigue, migraine, chronic pain, gastrointestinal disturbance, anxiety and depression.

#### 3.2.6 Complex regional pain syndrome (CRPS)

We identified 14 reports of possible CRPS (14), all of which occurred in females. The median age was 12 years (range 10–23 years), with a median onset interval of 1 day post-vaccination (range 0–82 days). In 12 cases (85.7%), 9vHPV was given alone. In three cases, symptoms localized to the vaccination arm (e.g., left upper limb numbness). 8 reports documented symptom onset in body regions unrelated to the vaccination site, including ankles, legs, spine, pelvic area, and upper limbs, often preceded by trauma (e.g., fractures, sprains) or physical activity. One report described symptom onset preceding vaccination, while other reports lacked necessary documentation.

#### 3.2.7 Primary ovarian insufficiency (POI)

We identified 21 reports that met the search criteria for POI (15), and 9vHPV was the only vaccine administered in these reports. Of these, 8 were diagnosed by a physician or medical documented as POI, and the other 13 reports were hearsay or did not contain enough information to be identified. No familial history of premature ovarian failure was documented in these cases.

#### 3.2.8 Acute disseminated encephalomyelitis (ADEM)

We identified 14 reports of possible ADEM (16): 8 were female, 5 were male, and 1 did not report gender. Among the reports reviewed, 12 were definitively diagnosed with ADEM, one was considered a suspected case of ADEM, and one case lacked sufficient information for a conclusive diagnosis. 9vHPV was administered alone in 12 cases (85.7%). All 14 reports were classified as serious.

#### 3.2.9 Transverse myelitis

We identified 19 reports that met the search criteria for transverse myelitis (17): 14 were female, 4were male, and 1 did not report gender. Of these reports, 15 were diagnosed as transverse myelitis, and 4 reports did not contain enough information to be identified. In 15 cases (78.9%), 9vHPV was given alone. 18 reports were classified as serious, while one was classified as non-serious.

### 3.2.10 Chronic inflammatory demyelinating polyneuropathy (CIDP)

We did not identify any reports of CIDP (18).

### 3.3 Data mining

Data mining is utilized to identify adverse events reported following the administration of 9vHPV that were disproportionately reported. Through disproportionality analysis, we quantified the reporting disproportionality of AEs associated with 9vHPV compared to other vaccines in the VAERS database. Of note, upon reviewing the prespecified conditions, we identified that six MedDRA PTs (anaphylactic shock, postural orthostatic tachycardia syndrome, dizziness postural, complex regional pain syndrome, premature menopause, acute disseminated encephalomyelitis) were disproportionally reported after 9vHPV (Table 3). Other prespecified outcomes, including GBS, CIDP and transverse myelitis, did not find any disproportionately reported terms. The characteristics of these reports have been described above.

**Table 3:** Data mining finding for pre-specified conditions to VAERS after 9vHPV administration, 2014–2024.

Prespecified conditions	PT	*N*	ROR (95% CI)
Anaphylaxis	Anaphylactic shock	45	2.91 (2.17–3.91)
POTS	Postural orthostatic tachycardia syndrome	106	9.03 (7.42–10.98)
POTS	Dizziness postural	36	1.9 (1.37–2.64)
CRPS	Complex regional pain syndrome	14	5.11 (3–8.71)
POI	Premature menopause	20	13.47 (8.51–21.3)
ADEM	Acute disseminated encephalomyelitis	14	2.42 (1.43–4.11)

ROR >1 indicates disproportionate reporting but not causation.

### 3.4 Comparison with pre-licensure safety data

A comparison of common adverse events between pre-licensure trials and the current VAERS surveillance is presented in Table 4. The most frequently reported non-serious events in both settings included injection site reactions (pain, swelling), headache, and dizziness. Syncope was also commonly reported in both datasets. The serious conditions of interest identified in this VAERS analysis (e.g., anaphylaxis, POTS, CRPS) were either not reported or reported at extremely low frequencies in the pre-licensure trials.

**Table 4:** Comparison of common and selected adverse events between pre-licensure clinical trials and post-licensure VAERS surveillance (first decade) for 9vHPV.

Adverse event (AE) category	Pre-licensure clinical trials data	Post-licensure VAERS data
Most common non-serious AEs	Injection site pain, injection site swelling, injection site erythema, and headache.	Syncope, dizziness, loss of consciousness, pallor, and injection site swelling.
Most common serious AEs	Pyrexia, allergy to vaccine, asthmatic crisis, and headache.	Headache, dizziness, fatigue, pyrexia, and pain.
Anaphylaxis	Reported as very rare events.	89 reports identified; 57 met Brighton criteria. Signal detected via disproportionality analysis.
POTS	Not reported in pivotal trial publications.	136 reports identified; 66 met clinical criteria. Signal detected via disproportionality analysis.
CRPS	Not reported.	14 reports identified, all in females. Signal detected via disproportionality analysis.
GBS	Not identified.	42 reports; 7 met Brighton criteria. No disproportionality signal found.
Mortality	5 deaths occurred across the clinical studies, No vaccine-related deaths.	57 death reports; majority lacked evidence of causal link.
Overall safety conclusion	Favorable safety profile; benefits outweigh risks.	Consistent with pre-licensure data; majority of reports are non-serious. Identified rare signals need further investigation.

9vHPV, 9-valent human papillomavirus vaccine; VAERS, Vaccine Adverse Event Reporting System; POTS, Postural Orthostatic Tachycardia Syndrome; CRPS, Complex Regional Pain Syndrome; GBS, Guillain-Barre syndrome.

Sources for Pre-licensure Data: U.S. Food and Drug Administration (3).

## 4 Discussion

We conducted a comprehensive appraisal of AEs reported to VAERS following 9vHPV administration during the first decade after its licensure (from December 2014 to December 2024). The safety profile observed in our review is consistent with evidence from pre-licensure clinical trials as well as other post-licensure monitoring and epidemiologic research (2, 3, 5, 19). During the analytic period, VAERS received 23,499 reports after 9vHPV. Overall, the majority were non-serious reports (92.5%) in the current analysis, compared with 97.4% for 9vHPV and 94.2% for 4vHPV reported in the previous study. The most commonly reported AEs (syncope, dizziness, headache, dizziness, and pain) were similar to those observed in previous 9vHPV and 4vHPV studies (5, 20). These findings reinforce the overall safety of 9vHPV and support its continued use in preventing HPV-related cancers and other diseases.

Syncope and dizziness were the most frequently reported AEs in both non-serious and serious reports, which is consistent with previous studies on HPV vaccines (21, 22). Syncope, in particular, is a well-known reaction to vaccination, especially among adolescents, and is often related to the stress or anxiety associated with the injection process rather than the vaccine itself. The high reporting rate of syncope underscores the importance of adhering to the ACIP recommendations to observe vaccine recipients for 15 min post-vaccination to prevent syncope-related injuries. Additionally, the predominance of headache and pain in serious reports highlights the need for healthcare providers to monitor these symptoms, as they may occasionally signal more severe underlying conditions.

We employed data mining and clinical review of reports as part of the post-licensure safety surveillance for 9vHPV. While the majority of AEs were non-serious, our analysis identified several rare but clinically significant conditions, including POTS, CRPS, POI and ADEM. These conditions have been the subject of public and scientific scrutiny, and our findings contribute to the growing body of evidence regarding their association with HPV vaccination. Our disproportionality analysis identified several MedDRA PTs that were disproportionately reported following 9vHPV vaccination, including anaphylactic shock, dizziness postural, POTS, CRPS, POI and ADEM. It is crucial to contextualize these statistical signals. Disproportionality in VAERS can arise from multiple factors, including a true causal association, but also from intensified media attention, stimulated reporting driven by public or professional concern, or reporting biases. Therefore, these signals are hypothesis-generating and do not imply causation; they highlight potential areas requiring further investigation through formal epidemiologic studies. The absence of disproportionate reporting for other prespecified conditions, such as GBS, transverse myelitis and CIDP, is reassuring and consistent with previous studies (23).

Among the signals, the high ROR for “premature menopause” (POI) (ROR = 13.47) warrants specific discussion given public attention to this issue. It is essential to interpret this finding in light of the very low absolute number of confirmed cases (*n* = 8) identified through clinical review against the background of millions of doses administered. Furthermore, none of these reports documented a familial history of premature ovarian failure. While the disproportionate reporting justifies continued scrutiny, the extremely rare occurrence and lack of supporting evidence from large, controlled studies to date suggest that, if a risk exists, it is exceedingly small. Similarly, for other signals like POTS and CRPS, evidence has not consistently demonstrated an increased risk following HPV vaccination (5, 24). Evidence of serious safety issues is currently conspicuously lacking, with existing temporally associated case reports either lacking biological plausibility or supported only by tenuous theoretical reasoning (24–26). Detection of positive signals does not definitively establish a causal link between vaccine administration and the reported adverse event (PT), but it can suggest potential safety signals warranting additional investigation.

This study has several strengths. First, it provides a comprehensive, decade-long overview of post-licensure safety data for 9vHPV, capturing a substantial volume of reports (*N* = 23,499) and enabling the assessment of trends and rare events over an extended period. Second, we applied standardized case definitions (e.g., Brighton Collaboration criteria for anaphylaxis and GBS) to enhance the clinical consistency and validity of case classification. Finally, our approach combined descriptive analysis, detailed clinical review of serious reports and deaths, and quantitative disproportionality analysis, offering a multi-faceted evaluation of potential safety signals. However, the limitations of VAERS data, including underreporting, reporting biases, and lack of an unbiased and unvaccinated comparison group, must be acknowledged. VAERS is a passive surveillance system, and the reported AEs do not necessarily imply causation. Additionally, the quality and completeness of reports vary, and many serious reports lack sufficient medical documentation to confirm diagnoses. The multiple comparisons conducted across numerous MedDRA Preferred Terms increase the possibility of identifying false-positive signals by chance alone. Despite these limitations, VAERS remains a valuable tool for detecting potential safety signals and generating hypotheses for further investigation.

## 5 Conclusion

This decade-long post-licensure safety surveillance of 9vHPV confirms its overall favorable safety profile, with the majority of reported AEs being non-serious and self-limiting. Disproportionality analysis identified several potential safety signals, including anaphylaxis, POTS, CRPS, POI and ADEM. These signals, derived from spontaneous reporting and subject to the biases inherent in such systems, warrant further investigation through formal epidemiological studies, such as cohort or case–control designs conducted within large, linked healthcare databases, to properly assess any potential causal associations. It is important to note that the serious events discussed were reported in absolute rarity relative to the extensive number of vaccine doses administered. Overall, the substantial benefits of 9vHPV vaccination in preventing HPV-related cancers and genital warts continue to far outweigh these potential risks. Continued pharmacovigilance and further rigorous epidemiological research remain essential to ensure and reinforce the ongoing safety of this vaccine.

## Data Availability

The original contributions presented in the study are included in the article/Supplementary material, further inquiries can be directed to the corresponding authors.

## Footnotes

1
https://vaers.hhs.gov/data.html
