# Supplementary material for: Post-licensure safety surveillance of 9-valent human papillomavirus vaccine using the vaccine adverse event reporting system, 2014–2024
Source: Front Public Health. 2026 Jan 20;14:1724482. doi: 10.3389/fpubh.2026.1724482 (PMC12864418; doi:10.3389/fpubh.2026.1724482)
Supplement: Supplementary file 1 [file Table_1.DOCX]

**Supplementary Table 1. MedDRA Preferred Terms used to identify prespeciﬁed conditions.**

| Prespeciﬁed conditions | MedDRA Preferred Terms |
| --- | --- |
| Guillain–Barre syndrome (GBS) | Guillain-Barre syndrome, Miller Fisher syndrome, demyelinating polyneuropathy |
| Anaphylaxis | Anaphylactic shock, anaphylactic reaction, anaphylactoid reaction, anaphylactoid shock |
| Postural orthostatic tachycardia syndrome (POTS) | Postural orthostatic tachycardia syndrome, dizziness postural, postural reﬂex impairment |
| Complex regional pain syndrome (CRPS) | Complex regional pain syndrome, mononeuropathy multiplex |
| Primary ovarian insufﬁciency (POI) | Premature menopause, ovarian disorder, amenorrhea |
| Acute disseminated encephalomyelitis (ADEM) | Acute disseminated encephalomyelitis, encephalomyelitis, noninfective encephalomyelitis, acute hemorrhagic leukoencephalitis, autoimmune demyelinating disease |
| Transverse myelitis | Myelitis transverse, myelitis, noninfectious myelitis |
| Chronic inflammatory demyelinating polyneuropathy (CIDP) | Chronic inflammatory demyelinating polyneuropathy |
| Death | Died |
